# The Effect of Necrotic Enteritis Challenge on Production Performance, Cecal Microbiome, and Cecal Tonsil Transcriptome in Broilers

**DOI:** 10.3390/pathogens11080839

**Published:** 2022-07-27

**Authors:** Gabriel Akerele, Walid G. Al Hakeem, Jeferson Lourenco, Ramesh K. Selvaraj

**Affiliations:** 1 Department of Poultry Science, The University of Georgia, Athens, GA 30602, USA; mgakerele@gmail.com (G.A.); walid.alhakeem@uga.edu (W.G.A.H.); 2 Department of Animal and Dairy Science, The University of Georgia, Athens, GA 30602, USA

**Keywords:** necrotic enteritis, microbiome, transcriptome

## Abstract

The objective of this study was to identify the effects of experimental necrotic enteritis (NE) infection on the production performance, gut microbiome, and cecal tonsil transcriptome in broiler birds. A total of 192 chicks were not-induced (control) or induced with NE. NE was induced by inoculating *Eimeria maxima* at 14 d of age and *Clostridium perfringens* at 19, 20, and 21 d of age. NE challenge increased (*p* < 0.01) NE lesion score at 7 days post-*E.maxima* infection (dpi), decreased (*p* < 0.01) average weight gain and increased (*p* < 0.01) mortality at 7 and 14 dpi. NE challenge increased (*p* < 0.05) gut permeability at 5, 6, and 7 dpi and increased ileal *C. perfringens* load at 5 dpi. NE challenge increased (*p* < 0.01) *Eimeria* oocyst shedding at 5, 6, 7, 8 and 14 dpi. NE challenge decreased (*p* < 0.05) the relative abundance of *Lactobacillaceae* and increased (*p* < 0.05) the relative abundance of *Campylobacteriaceae*, *Comamonadaceae*, and *Ruminococcaceae* at 6 dpi. NE challenge upregulated (*p* < 0.05) genes related to immune response and downregulated (*p* < 0.05) genes related to lipid metabolism at 6 dpi. It can be concluded that NE infection decreased beneficial bacteria and increased gut permeability.

## 1. Introduction

Necrotic enteritis (NE) is a *Clostridium perfringens*-induced disease of poultry and is caused by *Clostridium perfringens* type A strains and type C strains. Clinical and subclinical NE infections occur when *C. perfringens* colonizes and proliferates in the small intestine to produce extracellular toxins that damage the intestinal wall. Consumer concern over antibiotic resistance has increased pressure on poultry producers to increase the number of flocks raised without antibiotics. This recent push to remove antibiotics from poultry production has led to an increase in the incidence of NE in the poultry industry. The cause and problems associated with necrotic enteritis in broiler chickens are well documented [1,2,3].

The poultry industry seeks out alternative means of controlling pathogenic bacteria. The use of probiotics and natural growth promoters (NGP) has been receiving increased attention as potential alternatives due to their ability to alter intestinal microbial populations and improve host immunity and overall intestinal health. Several studies have explored the cecal microbiome [4] or jejunal microbiome [5] in chickens supplemented with antimicrobial alternatives that can ameliorate necrotic enteritis. Some other studies have assessed the expression of different genes at the transcript levels in birds supplemented with nutritional supplements designed to ameliorate necrotic enteritis [6]. However, despite the poultry industry’s attempts to mitigate NE through multiple approaches, NE continues to be a persistent problem in the poultry industry. It is therefore imperative to understand the pathology and molecular signature of NE. 

High throughput screening has become an increasingly important tool to study the gut microbiome and transcriptome of the host. A few studies have earlier attempted to assess the effects of necrotic enteritis on the chicken microbiome [7,8] and transcriptome [9,10]. Previous research has identified the role of the microbial community in altering gene expression in the ileum of the host [11] and thereby altering the susceptibility of poultry to necrotic enteritis. Ceca are the home to diverse microbial communities and have been the focus of microbiome studies [12]. Cecal tonsils are gut-associated lymphoid tissues that respond to infection by upregulating or down regulating transcription of different genes [13] and hence studying cecal tonsil transcriptome during NE challenge will quantify the host immune response during NE infection. To this end, high throughput screening has become an increasingly important tool. Understanding how the pathology, microbiology, and molecular signature of NE disease without supplementation can help will contribute positively to mitigation efforts against NE.

Therefore, the objective of this study was to identify the effects of NE on the production performance, gut microbiome, and cecal tonsil transcriptome in broiler birds induced with NE using an experimental model with *Eimeria maxima* and *C. perfringens* as described earlier [14].

## 2. Results

### 2.1. Effect of NE Challenge on Performance Parameters

From day 0 to 14 post-hatch, representing the period prior to infection, average feed intake, weight gain, and feed conversion ratio were comparable between non-challenged and challenged birds. On day 7 post-Eimeria infection, average weight gain was significantly decreased (*p* < 0.01) in challenged birds, while feed conversion and gut lesions were significantly higher (*p* < 0.01) in challenged birds compared to the control group. On day 14 post-Eimeria infection, bodyweight gain remained significantly decreased in challenged birds (*p* < 0.01) while the feed conversion ratio was comparable between challenge and non-challenged birds (*p* < 0.01, Table 1).

### 2.2. Effect of NE Challenge on Lesion Score

At 7 days post-Eimeria infection, gross lesions in the midgut of challenged birds were significantly higher than in unchallenged birds (*p* < 0.01, Table 2)

### 2.3. Effect of NE Challenge on Broiler Mortality

At 14 dpi, mortality was higher (*p* < 0.01) in the challenge group (12.5%) compared to nonchallenged birds (0%).

### 2.4. Effect of NE Challenge on Serum FITC-d Levels

**Figure 1:**
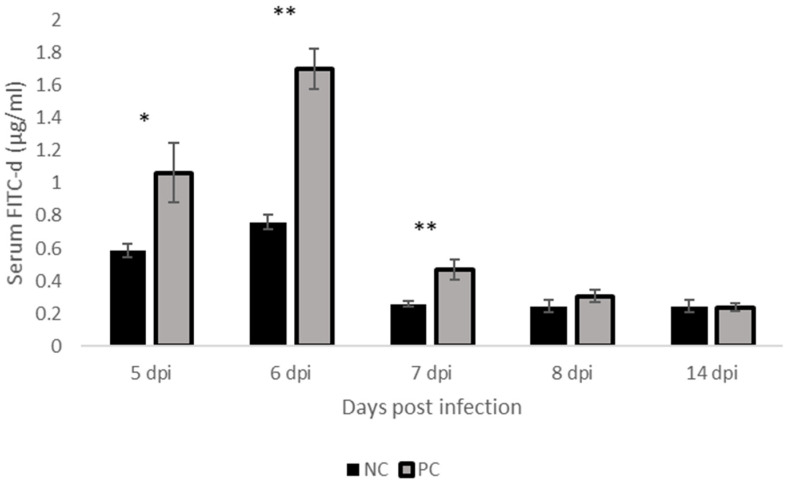
Effect of NE challenge on Serum FITC-d Levels. NC: Negative control; PC: positive control. Mean ± SEM of 8 replicates (n = 8) where ‘*’ (*p* < 0.05) or ‘**’ (*p* < 0.01) superscript differs significantly.

At days 5-, 6-, and 7-, post-Eimeria infection, Serum FITC-d levels were higher in challenged birds compared to non-challenged birds (*p* < 0.05, *p* < 0.01, Figure 1).

### 2.5. Effect of NE Challenge on C. perfringens Ileal and Cecal Colonization

**Figure 2:**
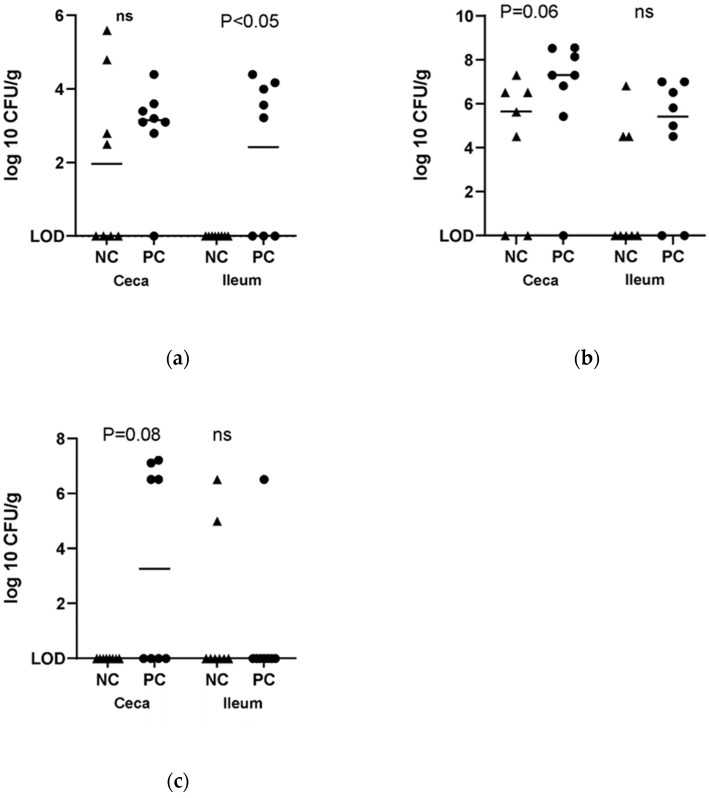
Effect of NE challenge on *C. perfringens* ileal and cecal colonization. (**a**) Ileal and cecal load at 5 dpi, (**b**) Ileal and cecal load at 6 dpi, (**c**) ileal and cecal load at 7 dpi. NC: negative control; PC: positive control. ‘ns’ indicates not significant.

On day 5 post-Eimeria infection, colonization of the ileum by *C. perfringens* was significantly higher in challenged birds (*p* < 0.05, Figure 2). On days 5-, 6-, and 8 post-Eimeria infection, colonization of the ceca by *C. perfringens* was statistically comparable between challenged and non-challenged birds (Figure 2). However, the cecal load of *C. perfringens* approached significantly higher levels (Figure 2) at 5 dpi.

### 2.6. Effect of NE Challenge on Eimeria Oocyst Shedding

**Figure 3:**
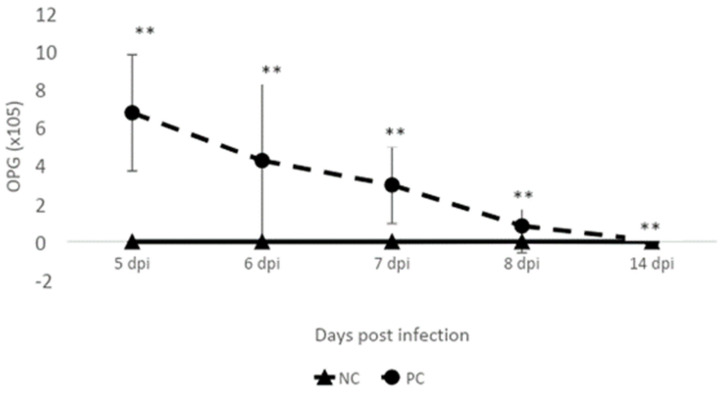
Effect of NE challenge on Eimeria oocyst shedding. NC: negative control; PC: positive control. Mean ± SD of 8 replicates (n = 8) where ‘**’ (*p* < 0.01) superscript differs significantly.

On days 5-, 6-, 7-, 8- and 14 post-Eimeria infection, challenged birds had significantly higher, although waning Eimeria oocyst output compared to unchallenged birds (*p* < 0.01, Figure 3).

### 2.7. Effect of NE Challenge on Cecal Microbiome

**Figure 4:**
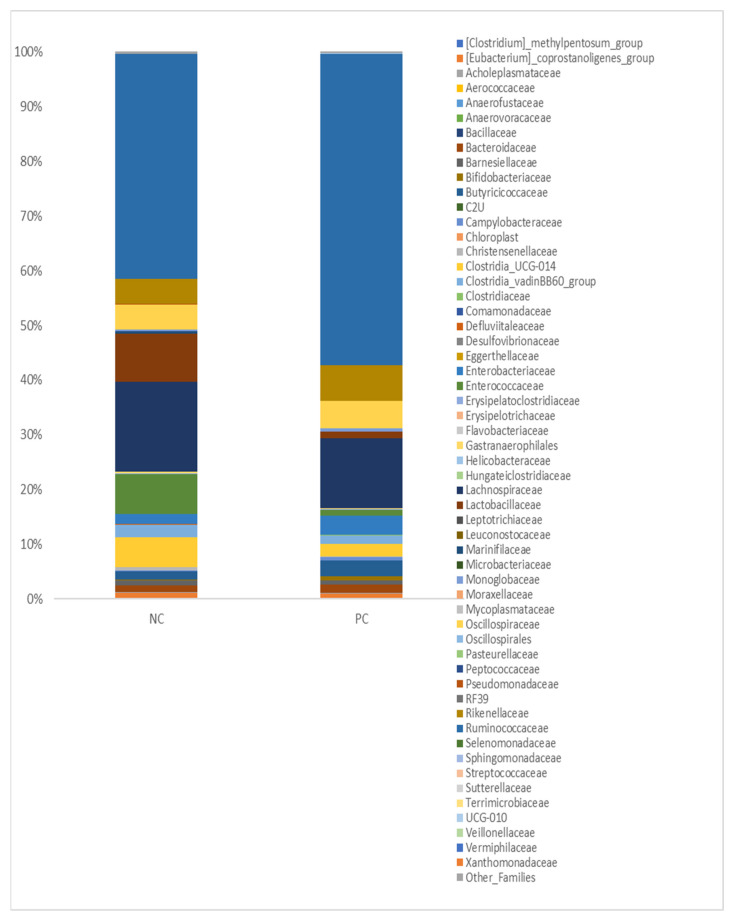
Relative abundance at the family level. NC: negative control; PC: positive control.

The relative abundance of 56 different families identified in the ceca of nonchallenged and challenged broiler birds is presented in Figure 4. The microbial profile was distinct between both groups, with members of the family of [Clostridium] methylpentosum group, Erysipelotrichaceae, Leptotrichiaceae, Mycoplasmataceae, and Pasteurellaceae being least abundant in the non-challenged group while members of the family Enterococcaceae, Lactobacillaceae, Lachnospiraceae, and Ruminococcaceae were among the most abundant members of the group. In challenged broilers, Leptotrichiaceae, Oscillospirales, Leuconostocaceae, and Helicobacteraceae were among the least abundant families, while Comamonadaceae, Monoglobaceae, [Eubacterium] coprostanoligenes group and Bacteroidaceae were the most abundant families (Figure 4).

### 2.8. Effect of NE Challenge on the Relative Abundance of Some Families

**Figure 5:**
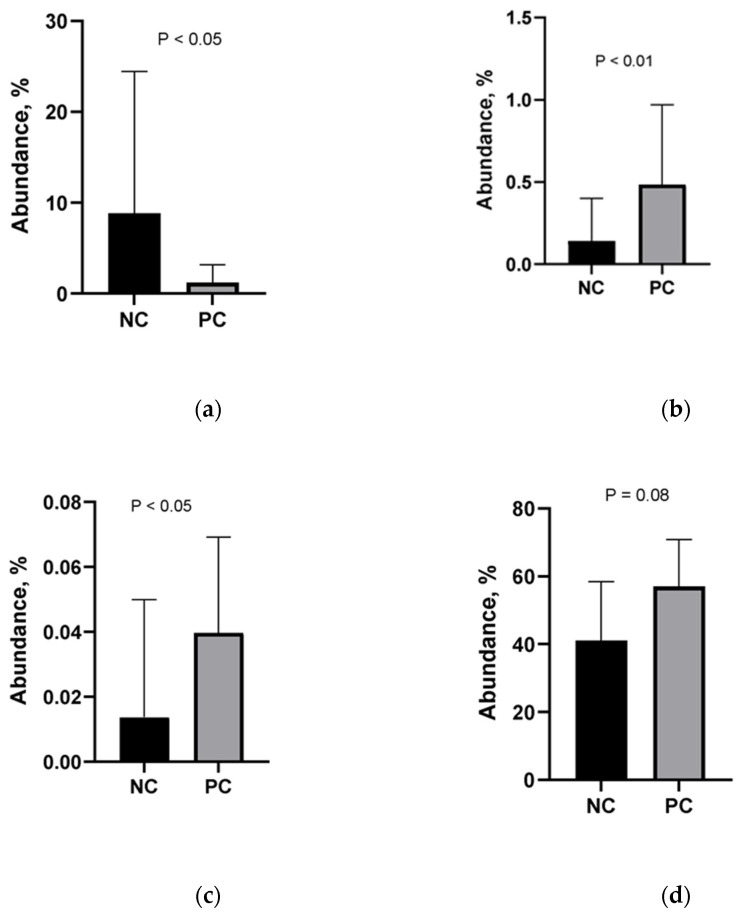
Relative abundance of Lactobacillaceae (**a**), Campylobacteraceae (**b**), Comamonadaceae (**c**), and Ruminococcaceae (**d**). NC: negative control; PC: positive control. Challenge: 5 × 10^3^ CFU *E. maxima* (d 14) + 10^8^ CFU *C. perfringens* (d 19–21). Error bars = SD of 8 replicates.

The relative abundance of members of the family of Lactobacillaceae was significantly lower in challenged birds (*p* < 0.05, Figure 5), while the relative abundance of members of the family of Campylobacteriaceae (*p* < 0.01, Figure 5) and Comamonadaceae (*p* < 0.05, Figure 5) significantly higher in challenged birds. The relative abundance of the members of the Ruminococcaceae was higher, although not statistically significantly (*p* = 0.08).

### 2.9. Effect of NE Challenge on Microbial Function Analysis

**Figure 6:**
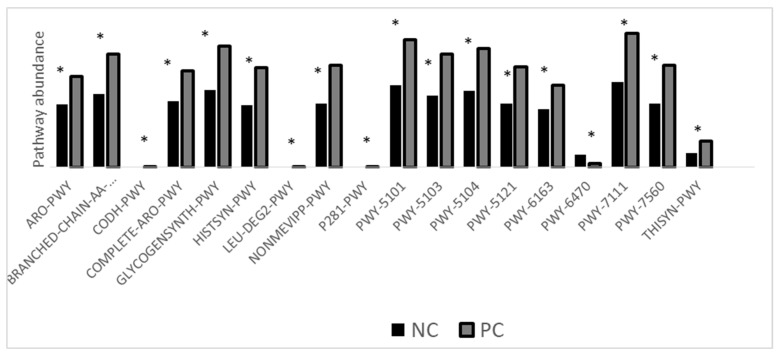
Microbial function analysis. NC: negative control; PC: positive control. Challenge: 5 × 10^3^ CFU *E. maxima* (d 14) + 10^8^ CFU *C. perfringens* (d 19–21). Error bars = SD of 8 replicates. ARO-PWY: Chorismate biosynthesis I. BRANCHED-CHAIN-AA-SYN-PWY: Superpathway of branched chain amino acid biosynthesis. CODH-PWY: Reductive acetyl-coenzyme A pathway I. Complete ARO-PWY: super pathway of aromatic amino acid biosynthesis. GLYCOGENSYNTH-PWY: glycogen biosynthesis I (from ADP-D-Glucose). HISTSYN-PWY: L-histidine biosynthesis. LEU-DEG2-PWY: L-leucine degradation I. NONMEVIPP-PWY: methylerythritol phosphate pathway I. P281-PWY: 3-phenylpropanoate degradation. PWY-5101: L-isoleucine biosynthesis II. PWY-5103: L-isoleucine biosynthesis III. PWY-5104: L-isoleucine biosynthesis IV. PWY-5121: Super pathway of geranylgeranyl diphosphate biosynthesis II (via MEP). PWY-6163: Chorismate biosynthesis from 3-dehydroquinate. PWY-6470: peptidoglycan biosynthesis V (β-lactam resistance). PWY-7111: pyruvate fermentation to isobutanol. PWY-7560: methylerythritol phosphate pathway II. THISYN-PWY: Superpathway of thiamine diphosphate biosynthesis I. 8 replicates (n = 8). ‘*’ (*p* < 0.05).

A total of 17 out of 18 pathways that were differentially expressed (*p* < 0.05, Figure 6) were upregulated in challenged birds. Only pathway 6470 was downregulated in challenged birds.

### 2.10. Effect of NE Challenge on Cecal Tonsils Gene Expression

There were 12 differentially expressed genes between challenged and non-challenged birds (*p* < 0.05, Table 3). A total of seven genes were up-regulated and five genes were down-regulated in challenged birds when compared to non-challenged birds. The greatest fold change in gene expression was a decrease in the Sodium Channel Epithelial 1 Subunit. The least change in expression was also a decrease in expression of a gene LOC12111069 whose function is not well understood.

## 3. Discussion

Necrotic enteritis can usually occur in an acute clinical form characterized by high mortality or in a chronic subclinical form, resulting in lower performance in broiler flocks [15]. In our study, the challenge model used induced acute disease [14,16,17], which manifested in a higher mortality rate in the challenged group compared to the control group. *Clostridium perfringens* infection resulted in significantly lower feed intake (*p* = 0.05), body weight gain (*p* < 0.01), and a significantly higher feed efficiency ratio (*p* < 0.01) in comparison with the control group at day 7 post*-Eimeria infection* post*-Eimeria* infection. This deterioration in the performance parameters was manifested in increased intestinal permeability (*p* < 0.05), which was significantly higher at 5-, 6-, and 7-days post*-Eimeria* infection. Oocyst shedding was also observed to decrease over time, with peak shedding at 5 days post*-Eimeria infection*. Research has previously shown that oocyst production peaks around 6 to 7 days post*-Eimeria* infection in coccidiosis [18]. The decline in oocyst shedding over time may be related to improving innate host defense mechanisms as the bird ages, such as increased production of Interferon gamma [19]. The intestinal interface forms a barrier between the environment and the internal milieu. A fully functional barrier is required to allow the absorption of nutrients and fluids and prevent the entry of pathogens and toxins [20]. Necrotic enteritis infection is well known to disrupt the intestinal barrier, which adversely affects performance [21]. *Clostridium perfringens* enterotoxin (CPE) is well characterized for its ability to bind/interact and modulate tight junctions [21]. Therefore, the higher load of *C. perfringens* in challenged birds at 5 days post-*Eimeria* infection may be associated with increased permeability as detected by increased serum FITC-D. The *C. perfringens* load in the ceca was similar in both groups at day 5 post-*Eimeria* infection. Interestingly, however, the trend appeared to gradually reverse by day 7 post-*Eimeria* infection, with higher cecal levels suggesting increased migration to the ceca. The presence of necrotic enteritis at the level of the ileum leads to gut dysbiosis, which in turn leads to increased oxygenation [22]. Following gut dysbiosis, a decrease in microbial diversity is observed mainly in obligate anaerobes with an increase in facultative anaerobes, particularly Proteobacteria [23]. The presence of inflammation and a disrupted gut leads to an increased flow of blood into the gastrointestinal tract (GIT), leading to the release of oxygen at the side of infection. The oxygenation of the GIT gives facultative anaerobes an ecological advantage to flourish and conversely restricts the presence of obligate anaerobes [22]. Obligate anaerobes are the main butyrate producers, which play an important role in maintaining gut homeostasis. The absence of these bacteria alters the regulation of the gut environment and results in lower signaling for nutrient absorption [24], which is reflected in the lower BW gain in the challenged group. Regarding oocyte shedding, it peaked as expected at 5 days post*-Eimeria* infection and decreased afterward [25]. 

Understanding the pathogen–host interaction during disease pathogenesis is crucial for the development of microbiome-based treatments. It is well documented that during NE manifestation, a shift in the microbiome occurs at the level of the ceca. However, it is still unclear whether this shift serves as a predisposing factor for NE, or it is a result of *Clostridium perfringens* proliferation [26]. The 16S rRNA sequences of the ceca on day 6 post-*Eimeria* infection revealed certain shifts in cecal microbiota. An eightfold decrease was observed in the lactobacillus species in the challenged group. Similar results were observed in other studies [27,28,29]. The decrease in the relative abundance of *Lactobacillus* species in the current study may be associated with lower performance parameters for the challenged birds. This is because *Lactobacillus* species are dominant throughout the GIT of healthy birds and play a vital role in poultry performance and immunity [30]. Lactobacillus species are competitive commensal bacteria that can exclude pathogenic bacteria in the host. They produce organic acids such as lactic acid and acetic acid, which reduce the pH in the host GIT [30]. This pH reduction produces a microenvironment suitable for microflora establishment and improves the ability of pathogens exclusion [30]. Lactobacillus species play an important role in immunomodulation in the GIT tract, decrease inflammation and improve performance parameters in their host.

An interesting interaction has been observed between *Campylobacter* spp. and *Clostridium perfringens*. *Clostridium perfringens* can utilize complex carbohydrates to generate organic acids utilized by *Campylobacter* spp. On the other hand, *Campylobacter* spp. acts as a hydrogen sink that increases *Clostridium* spp. fermentation capacity. Results from a previous study showed that the co-culture of *Campylobacter* spp. and *Clostridium* spp. improved their survivability following a mixed biofilm formation [31]. If such interaction occurs in vivo as well, it can provide an important insight into how a multispecies biofilm is a way to evade the immune system and how pathogenic bacterial communication plays a role in disease pathogenesis [32]. Our findings tended to confirm the previously mentioned results, given that the abundance of *Campylobacteriaceae* was approximately 5 times greater in the challenged birds than in the non-challenged ones. 

The relative abundance of *Comamonadaceae* increased in the challenged group. *Comamonadaceae* are a family of bacteria belonging to the beta-proteobacteria class that can utilize a wide range of organic compounds [33]. *Comamonadaceae*, a family within Proteobacteria, include *Comamonas* and *Acidovorax*, which are opportunistic pathogens capable of infecting multiple hosts such as plants [34], and humans [35]. An increase in the population of *Proteobacteria* has been previously reported with NE infection [36]. The increase in the relative abundance of such a pathogenic group indicates that the recovered birds are susceptible/carriers of pathogenic bacteria, which might lead to future issues concerning bird health and even food safety. 

It has been reported that the relative abundance of *Ruminococcaceae* bacteria decreases in the ceca following NE infection [26,37]. However, other studies reported increased *Ruminococcaceae* ileal relative abundance following NE infection [7]. Such contradiction in *Ruminococcaceae* relative abundance in different segments of the GI tract indicates that these microbial shifts are substantially different based on the gut segment [38]. *Ruminococcaceae* are butyrate producers, and an increase in their abundance may indicate increased production of butyrate; however, since we did not quantify butyrate in the current study, this conjecture cannot be verified. Moreover, a greater concentration of butyrate in the GIT of the challenged birds would likely contradict our other findings, such as the serum FITC-d levels and bird performance. Butyrate is an organic acid that can downregulate the production of proinflammatory cytokines by stimulating the production of IL-10 and TGF-beta [39,40]. The presence of butyrate in the ceca reduces the immune response against *C. perfringens*. Microbial functional analysis was carried out in the ceca at 6 dpi, representing the point of maximum gut permeability. The microbial functional analysis revealed several metabolic pathways that were significantly different following the *C. perfringens* challenge. These metabolic pathways might provide a novel insight into a drug target that might be utilized in the future to inhibit/kill *C. perfringens*. The ARO-PWY (chorismate biosynthesis I), Complete ARO-PWY (super pathway of aromatic amino acid biosynthesis), and PWY-6163 (chorismate biosynthesis from 3-dehydroquinate) were increased in the challenged group. Chorismate, which is the product of this metabolic pathway, serves as an intermediate for the biosynthesis of aromatic amino acids, folate cofactors, and siderophores [41]. These pathways are mainly utilized by Firmicutes, which explains their increased expression, as firmicutes increased following *C. perfringens* challenge. CODH-PWY (reductive acetyl-coenzyme A pathway I) was also higher following NE infection. This pathway, which is utilized by strict anaerobes, serves as a hydrogen sink that depletes the hydrogen and CO2 to produce Acetyl-CoA [42]. The depletion of hydrogen concentration increases the fermentation capacity of other bacteria, which might explain the increase of Firmicutes (main carbohydrate fermenter) following NE infection. Glycogen-synth-pathway increased in the challenged group. Bacterial species produce glycogen whenever excess glucose is in their surroundings. It has been hypothesized that the leaky gut and the damage that occurred at the level of the GIT facilitated the release of glucose into the ceca and stimulated the production of glycogen [43]. Like CODH-PWY, the L-leucine degradation I pathway was elevated. Both pathways produce acetyl-CoA, which serves as an energy source for a wide array of bacteria. Methylerythritol phosphate pathway (NONMEVIPP-PWY) I and (PWY-7560) II were increased in the challenged group, such pathway results in the production of phosphate-containing antigen that is recognized by γ-δ T lymphocytes [44]. The 3-phenyl-propanoate degradation pathway results in the production of pyruvate and fumarate as its product. Fumarate serves as an electron donor for *Campylobacter* spp. in strictly anaerobic conditions. The increase in fumarate production might have provided *Campylobacter* spp. with a suitable environment to proliferate, which was reflected by the increase in their relative abundance [45]. PWY-5101 (L-isoleucine biosynthesis II), PWY-5103 (L-isoleucine biosynthesis III), and PWY-5104 (L-isoleucine biosynthesis IV) are different pathways that result in the production of isoleucine by the residing microbiota and had greater expression in the challenged group. Isoleucine plays an essential role in Gram-positive bacteria adaptation to amino acid starvation in the host, and this may explain the increased expression of these pathways [46]. The super pathway of thiamine diphosphate biosynthesis I (THISYN-PWY), which results in the production of Thiamin diphosphate (vitamin B1), plays a role in energy metabolism. The increase in this pathway likely helped to support the bacterial proliferation at the level of the ceca. Peptidoglycan biosynthesis V (β-lactam resistance; PWY-6470) was the only down regulated pathway in this study in the challenged group. This pathway is utilized by *Enterococcus faecium* and other taxa. The downregulation of this pathway indicated that the population of *Enterococcus* and other critical groups were outcompeted by *C. perfringens* in the ceca following the necrotic enteritis challenge; and indeed, the challenged birds had an abundance of *Enterococcus* seven times greater than the control group (7.4 vs. 1.1%)

It was expected that a higher concentration of *C. perfringens* in the ceca would affect gene expression in the cecal tonsils. The sodium Channel Epithelial 1 Subunit Beta gene was upregulated in the challenged group, and this gene is responsible for neutrophil activation and mucus production. The increase in mucus production likely led to impairment of nutrient absorption and created a suitable environment for *Campylobacter* proliferation. This was manifested in the increased abundance of *Campylobacteriaceae* and decreased performance parameters in challenged groups. Furthermore, the VNN2 gene was upregulated in the challenged group. In humans, the VNN2 encodes a GPI-anchored cell surface molecule that plays a role in the transendothelial migration of neutrophils [47]. It is not clear if the increased expression of the VNN2 gene led to enhanced ingress or egress of neutrophils out of the cecal tonsils. However, the accumulation of neutrophils in blood vessels and vasoconstriction of blood vessels during an inflammatory response can produce anoxic conditions that support the growth of anaerobic bacteria. The upregulation of both CLCA1 and SCNN1B in challenged birds may be related to chloride and sodium transport, respectively. CLCA1 is an accessory protein of the calcium-activated chloride channel. Calcium ions are required for phospholipid recognition and hemolytic activity of a-toxin [48]. Aldo-keto reductase family 1, member B1-like, is involved in oxidative stress, cellular damage, and pathological change. An upregulation in this gene might have been associated with increased cell damage following the challenge. In agreement with increased gut permeability in challenged birds at 6 dpi, C1orf106, a colitis risk gene that regulates the stability of epithelial adherens junctions [49], was upregulated in challenged birds. However, a decrease in C1orf106 has been associated with increased gut permeability to solutes smaller than FITC-D in mice [50]. PDE9A is a phosphodiesterase 9A gene that is responsible for maintaining the homeostasis of cGMP. cGMP regulates a wide array of signaling pathways. These pathways are involved in the production of nitric oxide. Increased production of NOS is linked with cellular damage [51]. The Phospholipase A2 group IVE-like 2 (PLA2G4EL2) gene is involved in phospholipases, MAPK signaling pathway, and Ras signaling. *C. perfringens* TpeL toxin can block the RAS-pathway [52], which might explain the downregulation of the phospholipase A2 group IVE-like 2 gene in challenged birds. Moreover, *C. perfringens* toxins can cause ATP-depletion, which results in cell death. This may explain the downregulation of the ABCG-8 gene [53]. The ABCG-8 gene uses ATP-to translocate steroids and other lipids in the gut [54]). Stearoyl-CoA Desaturase (SCD) and Fatty Acid Desaturase 2 are involved in the production of acetyl-CoA, which is a vital component in lipid biosynthesis. Lipid biosynthesis is a key component in the synthesis of inflammatory responses. Downregulation of the SCD and Fatty Acid Desaturase 2 genes leads to an impaired immune response. Furthermore, decreased expression of the SCD-1 gene in challenged birds may have contributed to the depletion of cell membrane components. Increased gut permeability can increase bacterial translocation, which inhibits SCD-1. Inhibition of SCD-1 can lead to inflammation-associated sepsis [55]. The implication of the downregulation of F-Box and Leucine-Rich Repeat Protein 13 (FBXL13) in challenged birds is not clear. FBXL13 is an E3 ubiquitin ligase that targets CEP192 for ubiquitin-mediated degradation, thereby regulating microtubule nucleation activity and cell motility [56].

## 4. Materials and Methods

Experimental animals and Challenge: All animal protocols were approved by the Institutional Animal Care and Use Committee of the Southern Poultry Research Group, Athens, GA under experiment no. UGA0321. Birds were monitored daily by trained veterinary care staff for NE symptoms and euthanized using humane endpoints.

### 4.1. Birds and Housing

A total of 192 one-day-old male Cobb500 broiler chicks obtained from a commercial hatchery were raised in Petersime battery cages for 28 days at the Southern Poultry Research facility (Athens, GA, USA). Chicks were weighed by pen and then randomly distributed to one of two treatment groups: unchallenged and challenged birds, in a completely randomized design. A cage was treated as a replicate. Each treatment was replicated in eight cages (n = 8) of twelve chicks per cage. Birds were raised under standard management practices and had ad libitum access to a diet of mash feed and water. Feed intake, body weight, and mortality were measured weekly from the day of hatch. Mortality was recorded. Average feed intake and body weight gain (BWG) were corrected for mortality when calculating the feed conversion ratio (FCR) for each pen.

### 4.2. Effect of NE Challenge on Intestinal Lesions and Production Performances

The challenge model used was described earlier and demonstrated a severe effect on broiler performance under experimental conditions [14]. Birds in the challenge group were orally gavaged with 5 × 10^3^ oocysts of *E. maxima* on d 14 post-hatch and 1 × 10^8^ CFU/bird of a netB positive strain of *C. perfringens* on d 19, 20, and 21 post-hatch. On day 21 post-hatch, three birds per cage were randomly selected and scored for NE lesions on a scale of 0–3. The 0–3 scoring scale was as follows: 0 is normal, 1 shows slight mucus covering the small intestine, 2 has a necrotic small intestinal mucosa and 3 shows sloughed cells and blood in the small intestinal mucosa and contents.

### 4.3. Effect of NE Challenge on Gut Permeability 

Gut permeability was measured as described earlier [57] with modifications. Briefly, on days 5-, 6-, 7-, 8-, and 14- post-*E.maxima* infection, one bird from each cage was orally gavaged with 2.2 mg of fluorescein isothiocyanate dextran (FITC-d, MW 4000; Sigma-Aldrich, St Louis, MO, USA) in 1 mL of PBS. After 2 h, 3 mL of blood was collected and centrifuged at 1000× *g* for 15 min to collect serum. The FITC-d concentration in the serum was determined by measuring the amount of fluorescence in the serum using a spectrophotometer (Synergy HTX, microplate reader, Bio Tek Instruments, Inc., Winooski, VT, USA) at 485 nm excitation and 528 nm emission wavelengths. u. The amount of fluorescence in the samples was converted into FITC-d concentration based on a standard curve.

### 4.4. Effect of NE Challenge on C. perfringens Loads in the Ileal and Cecal Content

*C. perfringens* load in the ileal and cecal contents was measured as described earlier [58] with minor modifications. On days 5-, 6- and 8- post-*E. maxima* infection, whole ceca, and ileum from 1 bird/replicate were aseptically collected into stomacher bags and transported on ice to the laboratory. The digesta was flushed with sterile PBS until the digesta was removed from the gut tissue. Ileal and ceca were macerated in 3 times (wt/vol) PBS with a wooden mallet and homogenized for 1 min. A volume of 100 µL of each sample was serially diluted to obtain 1 × 10^−1^ to 1 × 10^−6^ dilutions. A volume of 10 µL of each dilution was plated in Tryptose soy cycloserine agar and incubated at 42 °C for 18–24 h. The colonies were counted and expressed as log10 CFU/g. Samples of presumptive positive colonies were confirmed using SyBr green qPCR and primers targeting the alpha-toxin and NetB gene.

### 4.5. Effect of NE Challenge on Eimeria Oocyst Shedding

Oocyst load in the fecal samples was measured as described earlier [59] using a salt-floatation technique. On days 5-, 6-, 7-, 8-, and 14- post-*E. maxima* infection, fresh fecal samples were collected from four spots from each cage. The fecal materials were ground, homogenized, and washed in 1× PBS. The oocysts were counted microscopically using a McMaster counting chamber. The number of oocysts per gram (OPG) of feces was calculated using the following formula:

OPG = 333.3 × oocyst count × dilution factor/sample weight.

### 4.6. Effect of NE Challenge on the Cecal Microbiome

On day 6 post-*Eimeria* infection, total DNA was extracted from cecal contents homogenates of one bird/cage using the Quick-DNA Fecal/Soil Microbe Zymo Research Kit. The total DNA was eluted in a final volume of 50 μL of elution buffer according to the manufacturer’s protocol and stored at −20 °C.

The total DNA extracted from samples was analyzed at the Georgia Genomics and bioinformatics core facility at the University of Georgia. The 16S rRNA gene was sequenced as described earlier [60]. Briefly, the V3-V4 hypervariable regions of the 16S rRNA gene were amplified using the primer FwOvAd_341f and ReOvAd_785r as described earlier [61] in a 25 μL reaction mixture. Each PCR reaction contained a DNA template (12.5 ng), 5 μL forward primer (1 μM), 5 μL reverse primer (1 μM), 12.5 μL 2× Kapa HiFi Hotstart ready mix (Anachem, Dublin, Ireland), and water to a final volume of 25 μL. The DNA was subjected to initial denaturation at 95 °C for 3 min. Amplification was then achieved by 25 cycles of denaturation at 95 °C for 30 s, annealing at 55 °C for 30 s, and extension at 72 °C for 30 s. The final extension was at 72 °C for 5 min. PCR products were cleaned using AMPure XP magnetic beads (Lab plan, Dublin, Ireland) and 80% ethanol. The PCR products were then submitted to another round of PCR to incorporate indexes (Illumina Nextera XT indexing primers, Illumina, Inc., San Diego, CA, USA) into the samples. Each PCR reaction contained 5 μL of each index primer, 25 μL 2× Kapa HiFi hot Start Ready-mix (Anachem, Dublin, Ireland), and 10 μL water. PCR cycling conditions were as previously described except for the number of amplification cycles, which was set to 8. PCR products were cleaned using AMPure XP beads and 80% ethanol, pooled, and paired ends were sequenced at a read length of 250 nucleotides on a MiSeq platform (Illumina, Inc., San Diego, CA, USA).

### 4.7. Effect of NE Challenge on Cecal Transcriptome

On day 6 post-*Eimeria* infection, cecal tonsils from 1 bird/cage were collected and stabilized in RNA later for 4 to 7 days. Excess RNA later was poured off, and the tissue samples were transferred to −80 °C. Total RNA was extracted using phenol-chloroform extraction [62]. The total RNA samples were submitted to the Georgia genomics and bioinformatics core facility at the University of Georgia for library preparation and sequencing.

Total RNA quantity and purity were assessed using a nanodrop (Thermo Fisher Scientific, Waltham, MA, USA), while RNA integrity was assessed using a bioanalyzer 2100 (Agilent, Santa Clara, CA, USA). The low throughput TruSeqLT was used to prepare 16 single-indexed, stranded libraries. Libraries were pooled and sequenced on the Illumina NextSeq 2000 with PE100 (Illumina, Inc., San Diego, CA, USA).

### 4.8. Effect of NE Challenge on Cecal Microbiome and Microbial Function Analysis:

The paired-end sequences were demultiplexed, converted to FASTQ files, and imported into QIIME 2 [63]. The non-biological nucleotides were removed, and sequences were denoised, dereplicated, and chimera-filtered using DADA2 [64]. Taxonomies were assigned to the sequences by using a pre-trained Naïve Bayes classifier trained on the SILVA 138 SSU database [65], and reads were classified by taxon using the fitted classifier [66]. Samples were rarefied to 141,432 sequences per sample prior to computing alpha and beta diversity. Phylogenetic Investigation of Communities by Reconstruction of Unobserved States (PICRUSt2) was performed to make inferences about the metabolic functions of the microbial community [67], and metagenome metabolic functions were assessed using the MetaCyc pathway database [68].

### 4.9. Effect of NE Challenge on RNA-Seq Analysis

Data were quality-assessed using FastQC software (ver. 0.11.8), and Trimmomatic [69] was used to quality-trim the raw read data. Only high-quality paired-end reads with a minimum length of 35 bases were retained for subsequent downstream mapping. The number of reads averaged around 24 million trimmed reads per sample and were aligned to the *Gallus gallus* genome using the STAR aligner ver. 2.7.9 [70]. Total mapped reads averaged 86%, and uniquely mapped reads averaged 73% across all samples, with the average number of uniquely mapped reads of 17.4 × 106 reads per sample. The HTSeq software [71] was used to extract the raw count data from each BAM file. Differential gene expression analysis was performed using two different packages, DESeq2 [72] and edgeR [73]. For DESeq2, following normalization of the raw data matrix, Wald’s test was employed for significance and determination of P-values, followed by a false discovery rate (FDR) adjustment. Results from edgeR were determined by Fisher’s Exact Test used for significance testing and determination of FDR values. Differential expression analysis was also carried out using QIAGEN CLC Genomics Workbench v.21.0.5 (Redwood City, CA). Only the gene IDs found to be significantly different between the 2 treatment groups (FDR *p*-value < 0.05) in both DESeq2 and edgeR were used in further analysis.

### 4.10. Statistical Analysis

Statistical analysis of performance: This was carried out with statistical software (SAS, v. 9.4, SAS Institute Inc., Cary, NC, USA). A cage was used as the experimental unit. Analysis of body weight gain (WG), feed conversion ratio (FCR), and feed intake (FI) was carried out using SAS PROC TTEST with a cage as an experimental unit (n = 8). Nonparametric analyses of mortality, gut lesions, and cecal colonization were carried out using Wilcoxon’s test. Significance was determined at *p* < 0.05. *p*-values between 0.05 and 0.1 were considered as approaching significance.

## 5. Conclusions

In conclusion, the NE challenge caused a shift in the microbiota and cecal tonsil transcription at 6 days post*-Eimeria* infection. *Campylobacter* was upregulated in the ceca of challenged birds. Unexpectedly, although *Clostridium* was numerically higher in the ceca of challenged birds, this was not statistically significant, which may be due to the time of sample collection. Most of the downregulated genes in the challenged birds are related to lipid metabolism, whereas the upregulated genes were related to immune response. Oocyst shedding and gut permeability were higher in challenged birds, which also showed decreased performance. Further studies are needed to compare the microbiome and transcriptome at multiple time points and in multiple tissues to get a complete picture of how chickens respond to infection.

## Figures and Tables

**Table 1:** Effect of NE challenge on performance parameters.

	D0-14			7 dpi			14 dpi		
	FI/kg	WG/kg	FCR	FI/kg	WG/kg	FCR	FI/kg	WG/kg	FCR
NC	0.51	0.18	2.833	0.45	0.28	1.607	0.99	0.70	1.414
PC	0.55	0.18	3.056	0.39	0.18	2.167	0.90	0.48	1.875
SEM	0.0046	0.0023	0.0063	0.0036	0.0043	0.0166	0.0092	0.0028	0.0063
*p*-Value	0.413	0.685	0.152	0.050	0.0005	0.0002	0.193	0.008	0.0075

NC: Negative control; PC: positive control. FI: feed intake.WG: average weight gain. FCR: feed conversion ratio. Challenge: 5 × 10^3^ CFU/mL *E. maxima* (d 14) + 10^8^ CFU *C. perfringens* (d 19–21). Mean ± Standard Error of Mean (SEM) of 8 replicates (n = 8).

**Table 2:** Effect of NE challenge on mid-gut lesion score at 7 days post-Eimeria infection.

Treatment	Lesion Scores				Rank Score Means	Chi Sq. *p*-Value
	0	1	2	3		
NC PC	24 7	0 13	0 2	0 2	4.50 12.5	<0.001

NC: Negative control; PC: positive control.

**Table 3:** Genes that were differentially expressed between NC and PC cecal tonsils.

Gene ID	Full Name	Mean	Log Fold Change	Direction (Challenged/Control)
SCNN1B	Sodium Channel Epithelial 1 Subunit Beta	153.1	−4.5	Up
PLA2G4EL2	Phospholipase A2 group IVE-like 2	50.6	1.8	Down
ABCG8	ATP Binding Cassette Subfamily G Member 8	17.7	1.8	Down
SCD	Stearoyl-CoA Desaturase	224.8	1.3	Down
VNN2	Vanin 2	437.9	−1.2	Up
CLCA1	Chloride Channel Accessory 1	61,693.7	−1.2	Up
LOC425137	Aldo-keto reductase family 1, member B1-like	2638.2	−0.8	Up
FBXL13	F-Box and Leucine Rich Repeat Protein 13	169.9	0.7	Down
PDE9A	Phosphodiesterase 9A	3320.2	−0.7	Up
C1orf106	Chromosome 1 Open Reading Frame 106 Pseudogene	308.6	−0.7	Up
FADS2	Fatty Acid Desaturase 2	865.1	0.7	Down
LOC12111060	N/A	535.4	−0.6	Up

Challenge: 5 × 10^3^ CFU *E. maxima* (d 14) + 10^8^ CFU *C. perfringens* (d 19–21). 8 replicates (n = 8).

## Data Availability

Not applicable.
